# FastMG: a simple, fast, and accurate maximum likelihood procedure to estimate amino acid replacement rate matrices from large data sets

**DOI:** 10.1186/1471-2105-15-341

**Published:** 2014-10-24

**Authors:** Cuong Cao Dang, Vinh Sy Le, Olivier Gascuel, Bart Hazes, Quang Si Le

**Affiliations:** VNU University of Engineering and Technology, Hanoi, Vietnam; Institut de Biologie Computationnelle, LIRMM, CNRS – Université Montpellier 2, Montpellier, France; Department of Medical Microbiology & Immunology, University of Alberta, Alberta, Canada; The Wellcome Trust Center for Human Genetics, Oxford University, Oxford, UK

**Keywords:** Amino acid replacement rate matrices, Maximum likelihood methods, Phylogenetic trees, Protein alignments, Large data sets

## Abstract

**Background:**

Amino acid replacement rate matrices are a crucial component of many protein analysis systems such as sequence similarity search, sequence alignment, and phylogenetic inference. Ideally, the rate matrix reflects the mutational behavior of the actual data under study; however, estimating amino acid replacement rate matrices requires large protein alignments and is computationally expensive and complex. As a compromise, sub-optimal pre-calculated generic matrices are typically used for protein-based phylogeny. Sequence availability has now grown to a point where problem-specific rate matrices can often be calculated if the computational cost can be controlled.

**Results:**

The most time consuming step in estimating rate matrices by maximum likelihood is building maximum likelihood phylogenetic trees from protein alignments. We propose a new procedure, called FastMG, to overcome this obstacle. The key innovation is the alignment-splitting algorithm that splits alignments with many sequences into non-overlapping sub-alignments prior to estimating amino acid replacement rates. Experiments with different large data sets showed that the FastMG procedure was an order of magnitude faster than without splitting. Importantly, there was no apparent loss in matrix quality if an appropriate splitting procedure is used.

**Conclusions:**

FastMG is a simple, fast and accurate procedure to estimate amino acid replacement rate matrices from large data sets. It enables researchers to study the evolutionary relationships for specific groups of proteins or taxa with optimized, data-specific amino acid replacement rate matrices. The programs, data sets, and the new mammalian mitochondrial protein rate matrix are available at http://fastmg.codeplex.com.

## Background

Amino acid replacement rate matrices represent the estimates of instantaneous substitution rates between amino acids. The rates simultaneously capture aspects of DNA-level mutation, the genetic code and protein-level selection strength, which varies based on similarity in chemical and physical properties. For example, we usually observe a high substitution rate between lysine and arginine (both positively charged and polar) and a low substitution rate between lysine and cysteine (neutral and nonpolar). Ideally, the replacement rate matrix parameters are optimized against the data under study, but in practice information content in typical sequence alignments is insufficient to do so. Instead, a small number of generic matrices are made available to researchers.

Amino acid replacement rate matrices are essential for many protein analyses, including estimating pairwise distances between protein sequences, or reconstructing protein phylogenetic trees using maximum likelihood or Bayesian frameworks[1, 2] and references therein. Amino acid replacement rate matrices can also be converted into score matrices for protein sequence alignment. Roles and applications of amino acid replacement rate matrices were summarized by Thorne[3].

A number of methods have been proposed to estimate the matrices from protein alignments since the time of Dayhoff[4]. These methods belong to either counting or maximum likelihood approaches. The counting methods are fast, but they are limited to only pairwise protein alignments and closely related amino acid sequences[4, 5]. The maximum likelihood methods have been designed to fully utilize the information contained in multiple protein alignments and the corresponding phylogenetic trees which must be estimated from the data[6–8]. This assumes that the trees are correct, which can be ameliorated by a Bayesian analysis over a set of plausible trees but this would increase an already large computational burden.

With the rapid rise in whole genome sequencing it is now increasingly common to have access to both large numbers of taxa and long concatenated sequence alignments. This creates the opportunity to estimate data-specific amino acid replacement matrices but also requires efficient computational methods because estimating amino acid replacement rate matrices from protein alignments by maximum likelihood methods is a complex and time-consuming process[7, 9, 10] and references therein.

Recently, a fully automated maximum likelihood estimation procedure was proposed and used to estimate matrices from different data sets[8, 10, 11]. It consists of two main steps: building maximum likelihood phylogenetic trees and estimating parameters based on the information contained in multiple protein alignments and the corresponding phylogenetic trees. Building maximum likelihood trees is itself a difficult problem because the number of possible trees increases exponentially with the number of sequences in the alignment[1, 12]. A number of maximum likelihood tree search heuristics have been developed to reduce the computational burden[13–16]; however, building maximum likelihood trees is still the most time consuming step in the estimation process. For example, in this study it took 98% (319 out of 324 hours) when estimating the amino acid replacement rate matrix from 100 large alignments in the HSSP (homology-derived structures of proteins) database[17].

In this paper, we propose a new maximum likelihood estimation procedure, FastMG, to work with large data sets. The key idea is to split large alignments into multiple non-overlapping sub-alignments with fewer sequences (each sub-alignment contains at most *k* sequences) in order to substantially reduce the computational burden of building maximum likelihood trees. The matrices are then estimated from the joint maximum likelihood analysis of the smaller sub-alignments instead of from the large original alignments.

A preliminary study showed that the splitting strategy significantly decreased the running time of the estimation procedure[9]. Here we demonstrate that a naïve random splitting method compromises the quality of the results. In contrast, our “tree-based splitting method” selects sub-trees that retain enough information to estimate accurate amino acid replacement rates. Experiments with different large data sets showed that the FastMG procedure yields similar quality matrices in much less time than the standard estimation procedure.

## Results and discussion

We assessed the performance of the FastMG procedure on three large data sets: HSSP data set[17], Pfam data set[18], and our concatenated protein alignment of mitochondrial proteins from 299 mammalian species with 3062 amino acid sites. The FastMG procedure was examined with the random splitting algorithm, the tree-based splitting algorithm, and different *k* values targeting sub-alignment sizes of 8, 16, 24, 32, and 64 sequences. All matrices were estimated on a personal computer (Intel 2.66 GHz, 8 GB RAM). The PhyML software version 3.0[14] was used to build maximum likelihood trees from alignments with options: 4 gamma categories model, no invariant sites, no bootstrap, SPR tree improvement, and JTT model. The XRATE software version 0.2[19] was used to estimate model parameters using information in the alignments and corresponding phylogenetic trees. Let us denote:

- FastMG^R^: The FastMG estimation procedure with the random splitting algorithm.
- FastMG^T^: The FastMG estimation procedure with the tree-based splitting algorithm.

: Replacement rate matrix estimated from data set M using the FastMG^R^ procedure and threshold *k* (e.g., is the matrix estimated by the FastMG^R^ procedure with *k* = 8).

: Replacement rate matrix estimated from data set M using the FastMG^T^ procedure and threshold *k* (e.g., is the matrix estimated by the FastMG^T^ procedure with *k* = 8).

- M^s^: Replacement rate matrix estimated from data set M using the standard maximum likelihood estimation procedure (e.g., HSSP^S^ is the matrix estimated by the HSSP data set using the standard estimation procedure).

We compared matrices in terms of both quality and running time. To avoid model bias due to over-fitting, each data set consisted of two alignment sets: the training alignment set and the testing alignment set. The matrices were estimated from alignments in the training set and subsequently used to build maximum likelihood trees for alignments in the testing set. Likelihood scores for test set alignments were used to compare the quality of different matrices as used in other studies[7, 8, 11]. Moreover, we used the Kishino-Hasegawa test[20] to assess the statistical significance of the difference between two matrices as used in previous studies[8, 11].

### HSSP data set

We selected 400 alignments from the HSSP database to assess the performance of estimation procedures. The 100 alignments with the largest number of sequences were used as the training alignment set for estimating matrices while the other 300 alignments were used as the testing alignment set. The 100 training alignments contained between 140 and 285 sequences (the mean and max pairwise distances between sequences were 0.481 and 1.286 respectively), for a total of 18325 sequences. The 300 testing alignments contained between 10 and 100 sequences (the mean and max pairwise distances between sequences were 0.635 and 1.846, respectively), for a total of 12854 sequences.

We examined the correlation between matrices estimated from the standard and the FastMG procedures. Table 1 shows high correlations between the 190 exchangeability coefficients of these matrices. As expected, the correlations increase with the increase of *k*. The results also show that tree-based splitting gives exchangeability parameters that are closer to the standard procedure. The opposite is true for the 20 frequency parameters of the matrices, likely because sub-alignments created by random splitting tend to represent the residue composition of the entire data set, whereas tree-based splitting gives sub-alignments that reflect the residue composition of individual clades. Another reason is likely because the random splitting algorithm selects taxa that are more distantly related and for which there was more time for the substitution process to reach equilibrium.

**Table 1:** **The Pearson correlations between the HSSP** ^**S**^ **matrix and other matrices estimated by the FastMG procedure**

Matrices	Frequencies	Exchangeability matrix
	0.992/0.986	0.989/0.991
	0.996/0.993	0.992/0.996
	0.998/0.995	0.994/0.997
	0.998/0.997	0.995/0.998
	0.999/0.999	0.997/0.999

The more important impact on tree likelihood values in pairwise comparisons between HSSP^S^ and other matrices estimated by FastMG^R^ and FastMG^T^ procedures are represented in Table 2. The matrices estimated from the FastMG^R^ procedure were not as good as the HSSP^S^ matrix for any *k* value. In contrast, the FastMG^T^ procedure generated high quality matrices and FastMG^T^ with *k* ≥ 16 was at least as good as the standard estimation procedure.

**Table 2:** **Pairwise comparisons between HSSP** ^**S**^ **and other matrices estimated from the FastMG procedure**

M1	M2	LogLK (M1-M2)	M1 > M2 (#TP)	M1 < M2 (#TP)	#M1 > M2 (#TP)	#M1 < M2 (#TP)
HSSP^S^		0.02	201 (130)	99 (65)	66 (27)	12 (4)
HSSP^S^		0.01	208 (126)	92 (64)	69 (26)	10 (4)
HSSP^S^		0.01	203 (114)	97 (72)	72 (26)	9 (5)
HSSP^S^		0.01	206 (119)	94 (59)	69 (24)	11 (4)
HSSP^S^		0.01	200 (101)	100 (63)	73 (16)	11 (4)
HSSP^S^		0.01	191 (124)	109 (75)	43 (19)	33 (15)
HSSP^S^		0.00	152 (95)	148 (81)	26 (5)	34 (9)
HSSP^S^		0.00	142 (88)	158 (89)	19 (4)	36 (11)
HSSP^S^		0.00	132 (78)	168 (87)	18 (3)	32 (6)
HSSP^S^		0.00	131 (72)	169 (80)	15 (4)	40 (4)

LogLK: the log likelihood difference per site between trees inferred using M1 and M2; a positive (negative) value means M1 is better (worse) than M2. M1 > M2: the number of alignments where M1 results in better likelihood value than M2; #TP: the number of alignments where tree topologies inferred using M1 and M2 are different. #M1 > M2 (*p* <0.05): the number of alignments where the Kishino-Hasegawa test indicates that M1 is significantly better than M2. #M1 < M2 (*p* <0.05): the same as #M1 > M2, but now M2 is significantly better than M1.

Table 3 shows the running time of the standard estimation procedure and the FastMG procedure with different splitting algorithms and *k* values for the HSSP data set. The estimation time of the FastMG procedure increased linearly with the increase of *k* (e.g., it took 11.0 hours and 22.9 hours to estimate and, respectively). This was an order of magnitude faster than the 323.7 hours needed to estimate HSSP^S^. Interestingly, FastMG^T^ was noticeably and consistently faster than FastMG^R^. The difference is not as large but suggests faster convergence for tree-based splitting.

**Table 3:** **The running time of the standard estimation procedure and the FastMG procedure with different splitting algorithms and** *k* **values for the HSSP data set**

Matrices	Building trees (hours)	Estimating parameters (hours)	Total time (hours)
	10.7/7.4	6.3/3.6	17.0/11.0
	22.9/19.0	6.0/3.9	28.9/22.9
	32.6/29.9	5.7/3.9	38.3/33.8
	42.3/40.0	5.5/3.9	47.8/43.9
	73.7/71.7	5.2/4.1	78.9/75.8
HSSP^S^	319.5	4.2	323.7

### Pfam data set

We also examined different estimation procedures on alignments from the Pfam database. The training alignment set contained the 100 largest alignments from the Pfam database with in total 7640 sequences and a range of 46 to 202 sequences per alignment (the mean and max pairwise distances between sequences were 1.341 and 62.428, respectively). The testing alignment set consisted of 480 other alignments with in total 5434 sequences and a range of 5 to 41 sequences per alignment (the mean and max pairwise distances between sequences were 1.174 and 63.096, respectively). Note that the Pfam alignments tended to contain fewer sequences than the HSSP alignments.

We observed similar values and trends as for the HSSP data set, with very high correlations between the Pfam^S^ matrix and matrices estimated from the FastMG procedure (see Table 4).

**Table 4:** **The Pearson correlations between the Pfam** ^**S**^ **matrix and other matrices estimated from the FastMG procedure**

Matrices	Frequencies	Exchangeability matrix
	0.994/0.990	0.993/0.993
	0.997/0.996	0.995/0.997
	0.998/0.999	0.995/0.999
	0.999/0.999	0.998/0.999
	1.000/1.000	0.999/1.000

The pairwise comparisons of tree likelihood values between the Pfam^S^ matrix and matrices estimated from the FastMG procedure are represented in Table 5. Again, we observed similar trends as for the HSSP data set. The quality of matrices increased with an increase of *k*, however, FastMG^R^ matrices were never as good as the Pfam^S^ matrix. In contrast, FastMG^T^ produced matrices that were in a majority of cases slightly better than the standard estimation procedure for all *k* values. For example, the matrix was better than the Pfam^S^ matrix on 290 out of 480 testing alignments. Moreover, the Kishino-Hasegawa test showed that the matrix was significantly better than the Pfam^S^ matrix on 104 testing alignments. The opposite was true only 41 times.

**Table 5:** **Pairwise comparisons between the Pfam** ^**S**^ **matrix and other matrices estimated by the FastMG procedure**

M1	M2	LogLK (M1-M2)	M1 > M2 (#TP)	M1 < M2 (#TP)	#M1 > M2 (#TP)	#M1 < M2 (#TP)
Pfam^S^		0.01	299 (67)	181 (41)	119 (8)	55 (8)
Pfam^S^		0.01	294 (54)	186 (35)	132 (6)	55 (3)
Pfam^S^		0.01	309 (57)	171 (38)	142 (10)	51 (3)
Pfam^S^		0.00	275 (40)	205 (35)	116 (6)	65 (2)
Pfam^S^		0.00	279 (38)	201 (28)	117 (4)	64 (3)
Pfam^S^		0.00	218 (54)	262 (64)	51 (3)	80 (8)
Pfam^S^		0.00	190 (33)	290 (51)	41 (2)	104 (6)
Pfam^S^		0.00	212 (33)	268 (39)	50 (2)	82 (1)
Pfam^S^		0.00	233 (36)	247 (36)	58 (1)	72 (0)
Pfam^S^		0.00	166 (21)	314 (31)	27 (0)	91 (2)

LogLK: the log likelihood difference per site between trees inferred using M1 and M2; a positive (negative) value means M1 is better (worse) than M2. M1 > M2: the number of alignments where M1 results in better likelihood value than M2; #TP: the number of alignments where tree topologies inferred using M1 and M2 are different. #M1 > M2 (*p* <0.05): the number of alignments where the Kishino-Hasegawa test indicates that M1 is significantly better than M2. #M2 > M1 (*p* <0.05): the same as #M1 > M2, but now M2 is significantly better than M1.

The running time of the standard estimation procedure and the FastMG procedure with different splitting algorithms and *k* values for the Pfam data set are represented in Table 6. We observed similar running time patterns as for the HSSP data set, however, the magnitude of the speed gain was less in this case (e.g. ~4 times for compared to Pfam^S^) because of the typically lower number of sequences in the Pfam alignments.

**Table 6:** **The running time of the standard estimation procedure and the FastMG procedure with different splitting algorithms and** *k* **values for the Pfam data set**

Matrices	Building trees (hours)	Estimating parameters (hours)	Total time (hours)
	2.8/2.4	5.5/3.1	8.3/5.5
	6.6/6.2	4.7/3.0	11.3/9.3
	9.9/9.4	4.2/2.9	14.1/12.3
	12.4/12.6	4.0/2.9	16.4/15.5
	20.8/22.4	3.4/2.8	24.2/25.2
Pfam^S^	35.8	2.9	38.7

### Mammalian protein alignment

Our mammalian protein alignment (Mam) consists of the concatenated 12 mitochondrial proteins (all except ND6) of 299 mammalian species and 3602 amino acid sites. The mean and max pairwise distances between sequences were 0.256 and 0.441, respectively. Because it is a single alignment, we used 10-fold cross validation to examine the performance of different estimation procedures[21]. In particular, the 3602 amino acid sites were randomly distributed across 10 non-overlapping partitions *P*_1_, …, *P*_10_ each consisting of 360 or 361 sites. The validation was repeated 10 times. Let *V*_*t*_ (*t* = 1…10) denote the *t*^*th*^ validation (i.e. the part *P*_*t*_ was used as the testing data and the other 9 parts were used as the training data).

Table 7 shows the likelihood comparisons between the Mam^S^ matrix and the matrices estimated from the FastMG procedure at the 10 validations. Again, the matrices estimated from the FastMG^R^ procedure were never as good as the Mam^S^ matrix, while the matrices estimated by FastMG^T^ with *k* ≥ 16 were similar or slightly better than the Mam^S^ matrix. For example, FastMG^T^ with *k* = 16 gave slightly better likelihood scores than the standard estimation procedure in all 10 validations and significantly better scores in 4 validations.

**Table 7:** **The log likelihood per site comparisons between the Mam** ^**S**^ **matrix and matrices estimated by the FastMG procedure**

					
LogLK per site	0.72/-0.04	0.58/-0.06	0.49/-0.05	0.42/-0.05	0.26/-0.02
# Significantly better	10/0	10/0	10/0	10/0	10/0
# Significantly worse	0/1	0/4	0/4	0/5	0/3

LogLK per site: average log-likelihood per site difference between Mam^S^ and the other matrices, positive/negative values indicate that the Mam^S^ matrix was better/worse than the other matrices. #Significantly better: number of tests where Mam^S^ is significantly better than the Mam^R^/Mam^T^ matrix (based on Kishino-Hasegawa test). #Significantly worse: number of tests where Mam^S^ is significantly worse than the Mam^R^/Mam^T^ matrix (based on Kishino-Hasegawa test).

We also examined the performance of the original MtMam matrix[22] and matrices estimated from the FastMG procedure. Table 8 shows that the matrices estimated by FastMG^T^ were significantly better than the original MtMam for all 10 validations.

**Table 8:** **The log likelihood per site comparisons between the original MtMam matrix and matrices estimated by the FastMG procedure**

					
LogLK per site	0.37/-0.39	0.23/-0.40	0.14/-0.40	0.07/-0.40	-0.09/-0.37
# Significantly better	0/0	0/0	0/0	0/0	0/0
# Significantly worse	0/10	0/10	1/10	4/10	7/10

LogLK per site: average log-likelihood per site difference between the original MtMam and the other matrix, positive/negative values indicate that the original MtMam matrix was better/worse than the other matrix. #Significantly better: number of tests where the original MtMam is significantly better than the Mam^R^/Mam^T^ matrix (based on Kishino-Hasegawa test). #Significantly worse: number of tests where the original MtMam is significantly worse than the Mam^R^/Mam^T^ matrix (based on Kishino-Hasegawa test).

The estimation time of different matrices is presented in Table 9. For this alignment with 299 species the FastMG procedure was an order of magnitude faster than the standard estimation procedure (e.g. FastMG^T^ with *k* = 16 was about 24 times faster than the standard estimation procedure). It also repeats the trend that the tree-splitting method is both faster and yields more accurate matrices.

**Table 9:** **The running time (hours) of different estimation procedures**

	Mam^S^					
Avg. time	22.2	0.5/0.4	1.5/0.9	2.2/1.4	2.7/1.9	4.3/3.6
Speed up		42/61.4	14.8/24.5	10.2/16	8.2/11.6	5.1/6.1

### Trends and special considerations

At the start of our studies we anticipated that alignment splitting would result in only minimal deterioration of matrix quality. Instead we observed, on balance, a small improvement in the performance of FastMG^T^ matrices compared to those obtained without splitting. Although the effect is small it is consistent and suggests that a systematic effect is at play. Although further studies are needed to better understand these effects, it is plausible that the specific elimination of deeper and often less well defined branches contributes to the effect. In addition, tree-based splitting results in alignments of more closely related sequences with lower risk of sequence alignment errors.

There were a number of alignments where the matrices inferred from the standard and FastMG^T^ procedures resulted in different tree topologies. This occurred in about 50-75% of cases for HSSP alignments and 10-25% of cases for the Pfam alignment (Tables 2 and5, columns 4 and 5). This different rate of occurrences is expected because the HSSP alignments have more sequences and are therefore more prone to topology differences. We considered the possibility that achieving a significantly better likelihood score was due to finding a different tree topology. However, in cases where the likelihood score is significantly better only a minority of alignments has a different tree topology.

Our three test cases all include a considerable amount of sequence divergence and all show that tree-splitting is superior to random splitting. A preliminary study on closely related influenza sequences showed that tree-splitting is still optimal[9]. However, in extreme cases the number of observed substitutions upon tree-splitting may become too small to be informative. In such a case random splitting may be preferred.

Another consideration is that our current FastMG procedure uses neighbor-joining to create the tree needed for the tree-based splitting algorithm. This scales well for alignments with hundreds or even thousands of sequences, but becomes inefficient for extremely large alignments. The performance of the FastMG procedure for such huge alignments will likely benefit from faster alignment splitting methods[23] and faster tree building methods (e.g. FastTree[24]) and this deserves further study if such cases become more common.

## Conclusions

Amino acid replacement matrices are essential for many statistical methods to analyze protein sequences. Maximum likelihood methods typically generate the best replacement matrices because they can fully utilize information in the multiple protein alignments. However, for this application maximum likelihood analysis is complex and computationally expensive. Here we propose a modified maximum likelihood procedure to estimate amino acid replacement rate matrices from large data sets. The key component of the modified estimation procedure is the splitting algorithm that divides large alignments into multiple sub-alignments with fewer sequences that are subsequently used to estimate matrices. The extensive experiments showed that the FastMG^T^ procedure performed well with large data sets and reduces the running time as function of the number of sequences from approximately quadratic to linear, as we expect based on the time complexity of tree inference (see Methods section). FastMG^T^ with *k* ≥ 16 was about an order of magnitude faster than the standard estimation procedure while it did not reduce the quality of estimated matrices.

Experiments strongly suggest that *k* = 16 is a good choice for the FastMG^T^ estimation procedure. Even the analysis of the 100 largest alignments of the HSSP database took less than a day with FastMG^T^ and *k* = 16 on a typical desktop computer. Thus, our method now enables researchers to avoid generic pre-calculated matrices and instead estimate optimal amino acid replacement matrices for their particular needs from large data sets on their personal computers.

## Methods

### Model

As usual, the amino acid substitution process is assumed to be independent among amino acid sites. Although the data might not be compositionally homogeneous across the sequences in the alignment (e.g., different amino acid compositions among the clades), we assume that the standard model for the amino acid substitution process over the tree is a Markov process with time-homogeneous, time-continuous, and time-reversible properties[6–8] and references therein. The model is represented by a 20 × 20 instantaneous substitution rate matrix *Q* = {*q*_*xy*_} where *q*_*xy*_(*x* ≠ *y*) represents the number of substitutions from amino acid *x* to amino acid *y* per time unit. The diagonal elements *q*_*xx*_ are assigned such that the row sums are all zero. Since the model is time reversible, the matrix *Q* can be decomposed into a symmetric exchangeability rate matrix *R* = {*r*_*xy*_} and an amino acid equilibrium frequency vector *Π* = {*π*_*x*_} such that *q*_*xy*_ = *r*_*xy*_*π*_*y*_ and

### Model estimation procedure

Given a set of *c* protein alignments *D* = {*D*_1_, …, *D*_*c*_}*,* the substitution model *Q* can be estimated by maximizing the likelihood *L*(*D*) using equation 1 as follows1

where *L*(*T*_*i*_, *ρ*_*i*_, *Q*; *D*_*i*_) is the likelihood of protein alignment *D*_*i*_ given phylogenetic tree *T*_*i*_; the rate variation model *ρ*_*i*_; and substitution model *Q*[7, 8].

Optimizing *L*(*D*) is a difficult problem because we have to simultaneously optimize parameters of *T*, *Q*, and *ρ*. Previous studies indicated that a good approximation of *Q (Q’)* can be obtained with near-optimal trees (*T’)* and rate variation model (*ρ*’)[7, 8, 10] and references therein*.* Because trees and rate variation models are computed a priori, the likelihood *L*(*D*) can thus be approximated by equation 2 as follows2

A better model *Q* can be estimated from alignments of *D* using an iterative approach as detailed in the 4-step standard estimation procedure as follows[8]:

**Standard estimation procedure**

- Step 0: Input a set of multiple alignments *D* and a starting matrix *Q* (typically input only exchangeability rate matrix *R*, the frequency vector *Π* is estimated from the data *D*).
- Step 1: Build phylogenetic tree *T*_*i*_ and rate across site model *ρ*_*i*_ for each alignment *D*_*i*_ using maximum likelihood tree construction programs such as PhyML[14].
- Step 2: Estimate a new exchangeability matrix *Q*’ using the approach described by Le and Gascuel[8] and the XRate software[19].
- Step 3: Compare *Q*’ and *Q*, if they are nearly identical, return *Q*’ as the optimal model. Otherwise, assign *Q* ← *Q*’ and goto Step 1*.*

Previous studies have showed that this estimation procedure usually stops after three iterations. The procedure is called “standard maximum likelihood estimation procedure”.

Building maximum likelihood trees in Step 1 is a computationally expensive problem[12]. Although a number of heuristics have been proposed for searching maximum likelihood trees[13–16], Step 1 is still the bottleneck of the estimation process. It is roughly estimated[25] that the computing time of PhyML (and of other similar maximum likelihood approaches) is in *O*(*n*^2^*s*), where *n* is the number of taxa and *s* the number of sites. Experiments with a number of different data sets have confirmed this approximation. Thus, it is expected that splitting the original alignments into two equally-sized sub-alignments divides the total computing time by a factor two. This property explains why the computing time to build trees displayed in Tables 3,6 and9 is approximately linear as a function of *k* (size of sub-alignments).

### Alignment-splitting algorithms

Consider a multiple alignment *D*_*i*_ of *n* sequences (*d*_*i*_^1^, …, *d*_*i*_^*n*^), splitting alignment *D*_*i*_ is a process to divide alignment *D*_*i*_ into non-overlapping smaller sub-alignments *D*_*i*_^1^, …, *D*_*i*_^*m*^ such that each sequence *d*_*i*_^*j*=1…*n*^ ∊ *D*_*i*_ belongs to exactly one sub-alignment *D*_*i*_^*t*^(*t* = 1…*m*). For example, alignment *D*_*i*_ of 8 sequences (*d*_*i*_^1^, …, *d*_*i*_^8^) can be split into three sub-alignments *D*_*i*_^1^ = (*d*_*i*_^1^, *d*_*i*_^2^, *d*_*i*_^8^), *D*_*i*_^2^ = (*d*_*i*_^3^, *d*_*i*_^4^, *d*_*i*_^5^), *D*_*i*_^3^ = (*d*_*i*_^6^, *d*_*i*_^7^). Let *k* be the maximum number of sequences in a sub-alignment; we seek a method of alignment splitting that allows us to minimize *k*, to maximize computational efficiency, while retaining adequate amounts of information in sub-alignments for estimating the amino acid replacement rates.

### Random splitting algorithm

Given a multiple alignment *D*_*i*_ of *n* sequences (*d*_*i*_^1^, …, *d*_*i*_^*n*^) and a threshold *k*, we describe a naïve splitting algorithm, called “Random splitting algorithm”. The general idea of the algorithm is to randomly split sequences (*d*_*i*_^1^, …, *d*_*i*_^*n*^) into sub-alignments such that each sub-alignment contains at most *k* sequences. To prevent creating too small sub-alignments that might not contain enough information for estimating the replacement rates, the algorithm also requires that each sub-alignment needs to contain at least *k*/2 sequences. Thus, each sub-alignment contains from *k*/2 to *k* sequences. The random splitting algorithm is fully described in Algorithm 1.

The random splitting algorithm is very simple; however, its main drawback is that sequences of the same sub-alignment might be very distantly related. This could compromise estimation of amino acid replacement rates.

### Tree-based splitting algorithm

**Figure 1:**
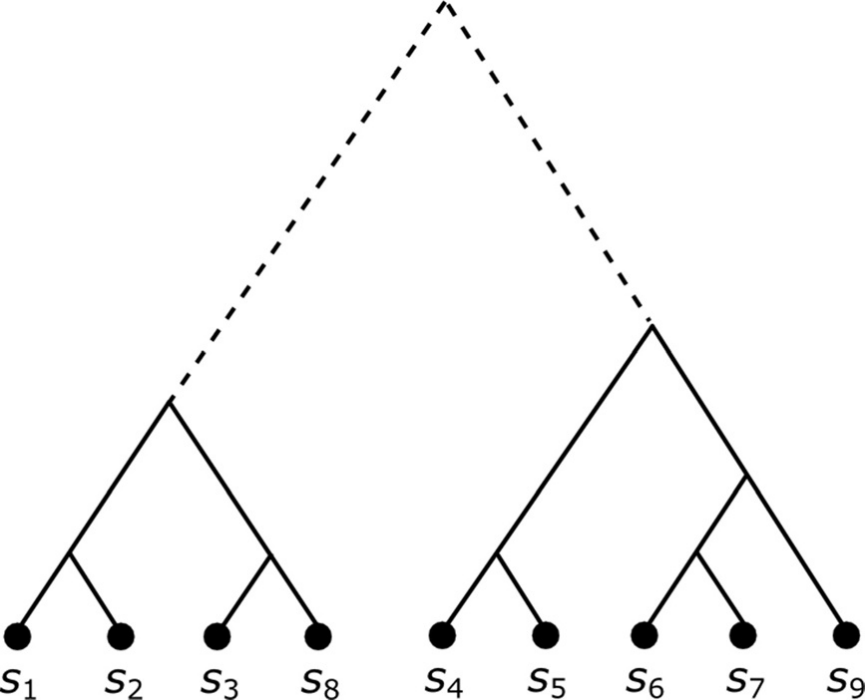
**Tree-based splitting example.** The tree-based splitting algorithm would divide this hypothetical tree for a 9-sequence alignment into two sub-alignments (*s*_1_, *s*_2_, *s*_3_, *s*_8_) and (*s*_4_, *s*_5_, *s*_6_, *s*_7_, *s*_9_), corresponding to the left and right sub-trees, respectively.

We designed a new splitting algorithm, called tree-based splitting algorithm, to maximize homology between sequences in each sub-alignment. Let *T*_*i*_ denote the phylogenetic tree relating sequences (*d*_*i*_^1^, …, *d*_*i*_^*n*^) of *D*_*i*_. The key idea of the tree-based splitting algorithm is that sequences in the same sub-tree of *T*_*i*_ should be split into the same sub-alignment. Figure 1 shows an example of 9 sequences related by a tree. The sequences can be split into 2 sub-alignments (*s*_1_, *s*_2_, *s*_3_, *s*_8_) and (*s*_4_, *s*_5_, *s*_6_, *s*_7_, *s*_9_) where (*s*_1_, *s*_2_, *s*_3_, *s*_8_) sequences belong to the left sub-tree while (*s*_4_, *s*_5_, *s*_6_, *s*_7_, *s*_9_) sequences belong to the right sub-tree.

The tree-based splitting algorithm follows the Neighbor-joining algorithm schema[26] to step-by-step group sequences into sub-alignments. The algorithm also requires that each sub-alignment contains at least *k*/2 sequences and at most *k* sequences. The tree-based splitting algorithm is fully described in Algorithm 2.

Note that the distance matrix between sequences used in the Neighbor-joining algorithm is estimated by the maximum likelihood method using the LG matrix[8]. Technically, we used BIONJ[27] (an improved version of the Neighbor-joining algorithm) to split large alignments.

### Fast maximum likelihood estimation procedure (FastMG)

The FastMG procedure consists of two phases: first, the large original alignments are split into non-overlapping sub-alignments by one of the alignment splitting algorithms; then the matrix is estimated by joint maximum likelihood analysis of the smaller sub-alignments instead of the large original alignments. The FastMG procedure is described by the following 5-steps

**Fast maximum likelihood estimation procedure (FastMG)**

- Step 0: Input a set of multiple alignments *D*; a starting matrix *Q* (typically input only exchangeability rate matrix *R*, the frequency vector *Π* is estimated from the data *D*); and a threshold *k*.
- Step 1: For each alignment *D*_*i*_ ∊ *D*, split *D*_*i*_ into sub-alignment *D*_*i*_^1^….*D*_*i*_^*m*^ using either the random splitting algorithm or the tree-based splitting algorithm.
- Step 2: Build phylogenetic tree *T*_*i*_^*j*^and rate across site model *ρ*_*i*_^*j*^ for each sub-alignment *D*_*i*_^*j*^ using maximum likelihood tree construction programs such as PhyML[14].
- Step 3: Estimate a new matrix *Q*’ from sub-alignments using the approach described by Le and Gascuel[8] and the XRate software[19].
- Step 4: Compare *Q*’ and *Q*, if they are nearly identical, return *Q*’ as the optimal model. Otherwise, assign *Q* ← *Q*’ and go to Step 2*.*
